# Optimization of Ultrasonic Flavonoid Extraction from *Saussurea involucrate,* and the Ability of Flavonoids to Block Melanin Deposition in Human Melanocytes

**DOI:** 10.3390/molecules25020313

**Published:** 2020-01-13

**Authors:** Chun-Yan Dai, Pei-Ran Liao, Ming-Zhuo Zhao, Chao Gong, Yue Dang, Yuan Qu, Li-Sha Qiu

**Affiliations:** 1 College of Life Science and Technology, Kunming University of Science and Technology, Kunming 650500, China; daichunyankm@126.com (C.-Y.D.); westpp@126.com (P.-R.L.); mingzhuozz@163.com (M.-Z.Z.); 18556589915@163.com (C.G.); dangyuki@126.com (Y.D.); 2 Yunnan Key Laboratory of Sustainable Utilization of *Panax Notoginseng*, Kunming 650500, China; 3 Laboratory of Sustainable Utilization of *Panax Notoginseng* Resources, State Administration of Traditional Chinese Medicine, Kunming 650500, China; 4 Kunming University of Science and Technology, Analysis and Testing Center, Kunming 650500, China

**Keywords:** *Saussurea involucrate*, ultrasonic extraction process, response surface, antioxidant, human melanoma A375 cells

## Abstract

(1) Background: Flavonoids are the primary medicinal ingredient of *Saussurea involucrate*, which have significant antioxidant capacity. Optimizing the extraction of *Saussurea involucrate* flavonoids (SIFs) and exploring the ability to block melanin deposition caused by reactive oxygen can greatly promote the development of *S. involucrate* whitening products. (2) Methods: Ultrasonic extraction process was optimized using the Box–Behnken design (BBD) and response surface methodology (RSM). Then, the effect of SIFs on antioxidant activity and anti-deposition of melanin, and genes related to the melanin synthesis are studied. (3) Results: The optimal extraction procedures are as follows: the extraction time, ethanol content, and solvent ratio (*v*/*w*) are 64 min, 54%, and 54:1, respectively. The reducing activity and scavenging rates of 2,2-diphenyl-1-picrylhydrazyl (DPPH), superoxide anion, hydroxyl radical, and ABTS^+^ were promoted as more *S. involucrate* flavonoid extract was added. The SIFs extract induced a decrease in the melanin synthesis by inhibiting the human melanoma A375 cell tyrosinase activity. SIFs also depress expression of melanin synthesis related genes. (4) Conclusions: the highest SIFs content was obtained by using 54% ethanol and 54:1 solvent ratio (*v*/*w*) for 64 min. The extract of SIFs exhibited good ability of antioxidant and anti-deposition of melanin in human melanocytes.

## 1. Introduction

*Saussurea involucrate* (Kar. et Kir.) Sch.-Bip. belongs to the *Asteraceae* family of the genus *Saussurea*, which is used to treat rheumatoid arthritis and regulate the menstrual cycle [1]. Pharmacologic studies show that many medicinal values of *S. involucrate* are derived from its flavonoids, and these include anti-neoplastic [2], anti-arthritic [3], anti-oxidative, anti-aging [4,5], and anti-fatigue [6] properties. Flavonoids also play an important role in blocking melanin deposition and skin whitening products. Previously, we found that the extracts of flavonoid from *Panax notoginseng* stem and leaf had a good inhibitory effect on tyrosinase activity and melanin production in B16 cells [7]. Kudo et al. [8] reported that flavones from *Scutellaria baicalensis Georgi* could inhibit melanogenesis and intracellular melanosome transport in B16F10 cells. But it is unknown whether the *S. involucrate* flavonoids (SIFs) possess a whitening function, which could allow SIFs to become a main raw material of whitening products.

Reducing melanin synthesis and pigmentation is the goal of whitening products. Tyrosinase is a major rate-limiting enzyme in melanin formation. Moreover, tyrosinase activity is regulated by free radicals, and thus, the melanin synthesis could be blocked by enhancing antioxidation. In vitro antioxidant study is a method to evaluate antioxidant capacities of plants, which is widely used in many fields, such as food, health products, drugs and cosmetics, etc. [9].

An effective method for the extraction of effective compounds from natural products is through ultrasonic extraction, which can make the extract continue to shock, contribute to the solute diffusion, improve the extraction rate of total flavonoids and the use of raw materials, which is the relatively new method for flavonoids extraction. Compared with the heated extraction method, ultrasonic extraction can reduce extraction time and increase extraction rate [10]. The response surface methodology (RSM) has been widely used for process optimization and the Box–Behnken design (BBD) is one statistical model of the response surface design methods. BBD represents an independent quadratic design that does not contain an embedded factorial or fractional factorial design. Compared with other design methods, BBD is easy to design and to analyze statistically [11]. It can reduce the number of experimental runs, cost, and time. Therefore, it is widely used in the extraction process optimization of flavonoids, and can obtain a better technological condition to extracting total flavonoids from *S. involucrate* by using a BBD of response surface methodology.

This study, for the first time, optimized the SIFs ultrasonic extraction processing technology and explored if SIFs could inhibit melanin synthesis in human melanoma A375 cells.

## 2. Results and Discussion

### 2.1. Optimization of the SIFs Extraction

Three major factors which included time (A), ethanol content (B) and solvent ratio (*v*/*w*) (C) were selected (Table 1). The proper ranges of these three factors were selected by single factor experiment on the bases of flavonoids content (Supplementary Figure S1), and the content of flavonoid was defined as the response value *Y*.

The designed matrix, experimental values, and predicted values are shown in Table 2. Multiple regression analysis of these experimental results was conducted using Design-Expert Software. The relationship between the response variable and the independent variables was expressed according to the equation:

Y = −33.37 + 0.33A + 0.69B + 0.29C − 0.0021AB + 0.0025AC − 0.0030BC − 0.0027A^2^ − 0.0037B^2^ − 0.0024C^2^ (1)

where Y represented the SIFs content, and A, B, and C represented time, ethanol content, and solvent ratio, respectively.

### 2.2. Analyses of the Regression Coefficients and Variances

**Figure 1:**
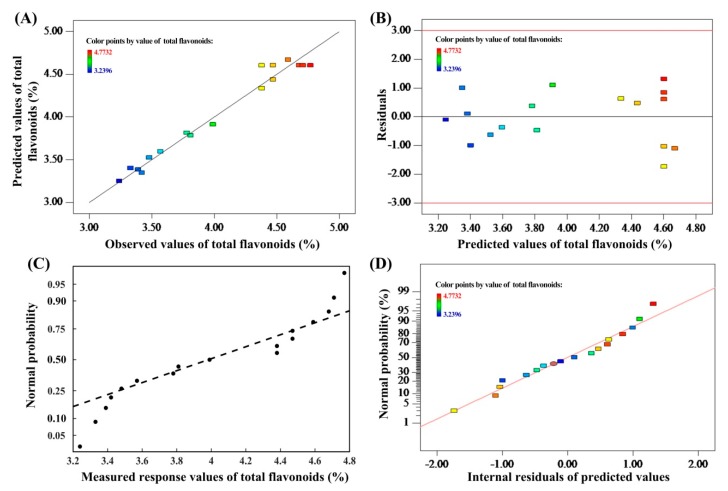
Plot of predicted vs observed values of the total flavonoids (**A**); the plot of the residuals versus the predicted response (**B**); the normal probability for the measured response values (**C**); Normal probability plots of residuals (**D**).

The ANOVA test (*F*-test) was used to check the statistical significance of the second-order polynomial Equation (1). The *F*-test showed that the *F* value of the second model was as high as 25.8661, and the values of “Prob > F” less than 0.0500 indicate model terms are significant. This shows that the model is very suitable for experimental data. The adjusted *R*^2^ (*R*^2^_Adj_) showed that the sample variation of 0.9333 for the SIFs content can be attributed to the independent variable. The quadratic regression model (*R*^2^ of 0.9708) showed that the actual results fit well with the predicted results by the model, which is verified in Figure 1A [12]. Therefore, the curves of the experimental and predicted values are linear [13]. A relatively low coefficient of variation (3.5499%) illustrated that further experiments were feasible with good accuracy and reliability [14]. The coefficient estimates of the second-order polynomial equation (7), with the corresponding *p*-values, are presented in Table 3.

Furthermore, the internal residuals of the model were randomly distributed (Figure 1B), so the uniformity of the residuals variance were consistent with the optimization requirements [15]. In addition, the fitted curve of the residuals was linear, indicating that the measured response values and internal residuals were normally distributed (Figure 1C,D) [13].

What is most needed is an insignificant lack of fit, because a significant lack of fit shows that the regression response relationship is not considered in the model [16]. In the study, the *p*-values showed the lack of fit at 0.7866 (more than the 0.05, confidence level is 95%), which meant that the lack of fit in this model was insignificant (Table 3), and the response value in the model fit well with the experimental results (Figure 1). It could be concluded from the above results that the RSM developed in this study predicted SIFs contents satisfactorily.

### 2.3. Analysis of the RSM

To optimally extract conditions, RSM plots provide a method to visualize the relationship between the response value and factor level. Table 3 and Table 4 inferred that A^2^, B^2^, and AC were the strongest variables regarding the operating efficacy of SIFs concentrations with a *p*-value < 0.0001, 0.0011, and 0.0012, respectively. The coefficient estimates were −0.61, −0.37, and 0.38, respectively. In this model, the *p*-values and coefficient estimates (as tools) were used to check significance and the efficacy of each coefficient, which, in turn, could indicate the type of interaction between the variables. Thus, the lower the *p*-value, the higher the absolute value of the coefficient estimate, which made the corresponding coefficient effect more significant [17].

**Figure 2:**
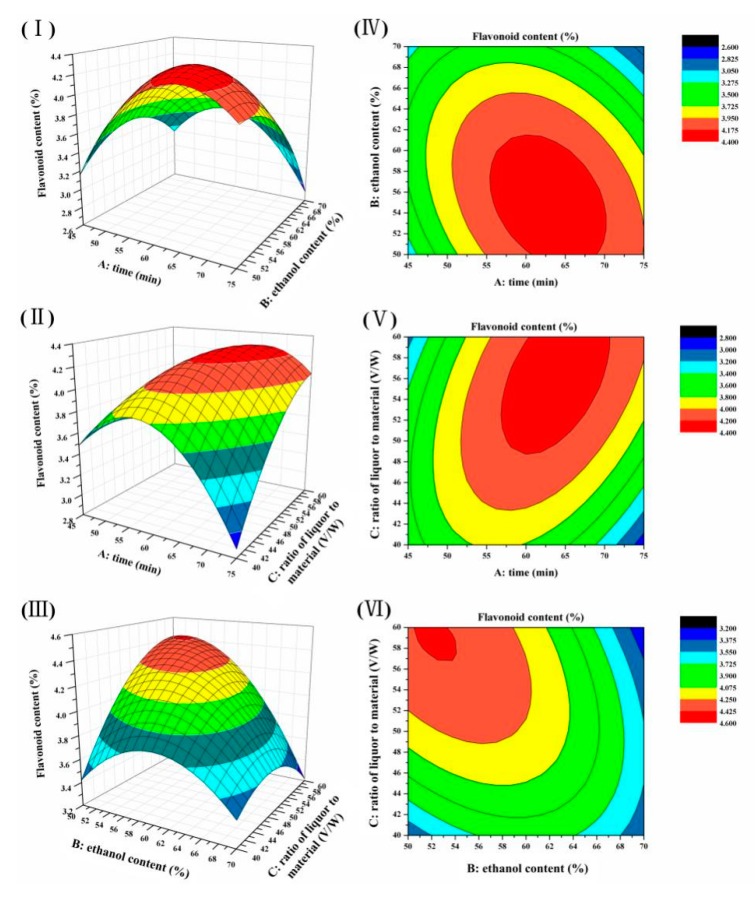
Response surface plots. (**I**–**III**) were showing the effects of extraction time (A), ethanol content (B), and (C) ratio of liquor to material on the content of total flavonoids, respectively. (**IV**–**VI**) were their contour plots, respectively.

A 3D surface graph (Figure 2I–III) of the total flavonoid content and the contour curve (Figure 2IV–VI) of the two test variables were generated from the final model to describe the interaction between the independent variables and the optimal process parameters. Each graph was completed with other factors were kept each time at their respective zero levels. If the contour plot has a circular shape, the interactions between the corresponding factors are negligible. The elliptical shape of the contour plot indicated that the interaction between the variables contributed to the content of total flavonoids at a significant level.

Based on the data shown in Table 3 and Figure 2, the ranking of the interaction effect in the model between the independent variables from high to low is AC > AB > BC. The effects of A (time) and C (liquid to material ratio) on the SIFs content are shown as a 3D-plot and the associated contour plot. The elliptical shape of the contour plot illustrates a significant (*p* = 0.0012) correlation between A and C, which contributes to the different SIFs contents. The SIFs contents increase when the time increased from 45 to 64 and the solvent ratio increased from 40:1 to 55:1 (Table 3; Figure 2II,V). What’s more, the SIFs content was greater than 4.67%, when the ethanol content increased from 50% to 56%, and the extraction time increased from 45 and 64 min (Figure 2I,IV). In addition, the interaction between B (ethanol content) and C also has a significant effect on the flavonoids content (*p* = 0.0041) (Table 3; Figure 2III,VI). In this study, it was most important that the extraction technology was economical and feasible in whole extraction process, and could reduce the cost and time of mass production in future.

### 2.4. Validation of the Model

The predicted optimum parameters for obtaining a high SIFs content were as follows: an extraction time of 63.93 min, an ethanol content of 54.21%, a solvent ratio (*v*/*w*) of 54.32:1, and a maximal response of 4.80% as predicted by the model equation. However, this optimal condition could be modified in actual production as follows: an extraction time of 64 min, and ethanol content of 54%, and a solvent ratio (*v*/*w*) of 54:1. Under the optimum conditions, the average SIFs yield was 4.89% ± 0.54% (n = 5), which showed no significant difference from the predicted value. The results showed that the regression model could accurately predict the extraction of SIFs.

Ultrasonic extraction of flavonoids from plants is widely applied of *Clerodendrum cyrtophyllum* Turcz [18], *Sparganii rhizome* [19], and *Aconitum gymnandrum* [20]. Compared with the traditional method of heat extraction, ultrasound promotes the penetration of solvents into plant raw materials, and releases intracellular products by destroying the cell wall [21], thereby increasing extraction efficiency and shortening extraction time.

### 2.5. SIFs Extract Antioxidant Activities

**Figure 3:**
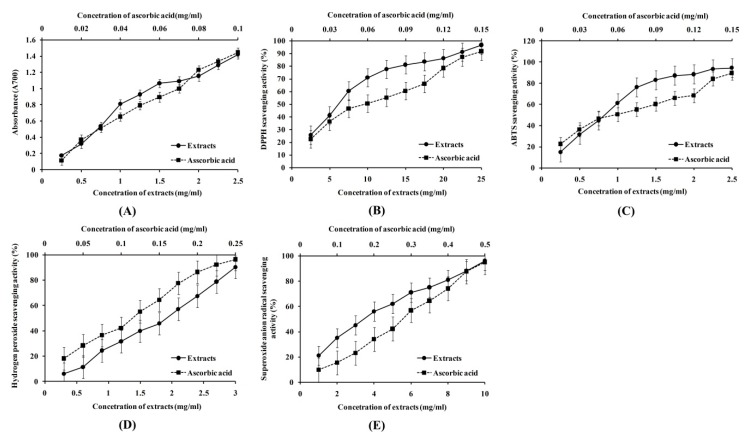
Antioxidant activities of ascorbic acid and the *Saussurea involucrate* flavonoids (SIFs) extract. (**A**–**E**) were the ferric ion reducing activity, DPPH radical scavenging rate, ABTS^+^ radical scavenging rate, hydroxyl radical scavenging rate and superoxide anion scavenging rate, respectively.

The reduction ability of SIFs extract was evaluated by measuring the conversion of Fe^3+^ to Fe^2+^. Generally, the SIFs extract shows a dose-dependent reducing power at 700 nm [22]. The extract reducing power (Figure 3A) and ascorbic acid concentrations increased with the extract concentrations; however, even though they were all positively correlated, significance was not obtained (multiple R were 0.976 and 0.995, respectively; *p* > 0.05 respectively). The EC_50_ values of the extract and ascorbic acid were 0.69 and 0.032 mg/mL, respectively (these concentrations were taken at an OD_700_ = 0.5).

Moreover, DPPH reducing power, DPPH scavenging activity, the formation of the superoxide radical, hydrogen peroxide, and ABTS^+^ are the 5 most common antioxidant indices measured, in vitro [9]. In this study, as shown in Table 5 and Figure 3B–E, DPPH, ABTS^+^, hydrogen peroxide, and superoxide anion radical scavenging activities of SIFs and ascorbic acid were increased significantly with increasing concentrations. There were significant correlations between the scavenging activities and extract concentrations (multiple R were 0.935, 0.945, 0.995, and 0.989, respectively; *p* < 0.05). There were also significant correlations between the scavenging activities and ascorbic acid measurements (multiple R were 0.990, 0.984, 0.996, 0.997, respectively; *p* < 0.05). The EC_50_ of the extracts were 6.13, 0.88, 1.8, and 3.84 mg/mL, respectively, which was higher than the EC_50_ of ascorbic acid (0.063, 0.065, 0.11, and 0.27 mg/mL), respectively.

### 2.6. The Effect of SIFs Extract on A375 Cell Activity

**Figure 4:**
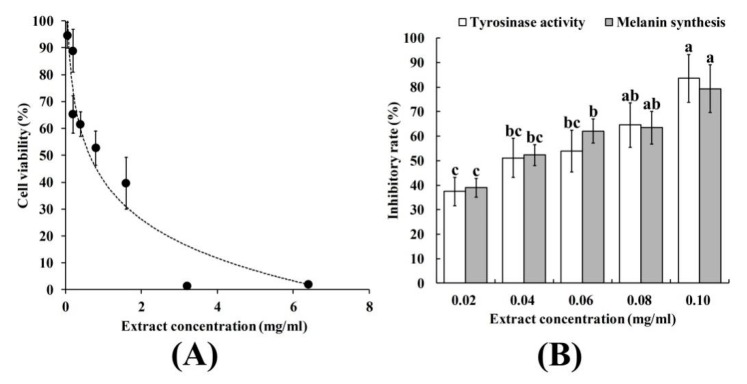
The effect of SIFs extract on cell activity (**A**) and inhibition rate of tyrosinase and melanin synthesis in melanoma cells A375 (**B**). Different small letters mean significant differences at *p* < 0.05.

The safety of the SIFs extract is extremely important in the investigation of cell viability, cellular tyrosinase activity and melanin level in A375 cells. The results showed that the cell viability was still about 85% at a 0.12 mg/mL SIFs extract concentration (Figure 4A). Thus, SIFs extract concentrations below 0.12 mg/mL could be adopted for subsequent experiments in the study.

Tyrosinase activity and melanin production were significantly reduced by extract in a dose-dependent manner (Figure 4B), and the inhibition ratio was positively correlated with extract concentration (multiple R were 0.979 and 0.962, respectively; the *p*-values were 0.00586 and 0.0113, respectively), and the IC_50_ was 0.13 and 0.10 mg/mL, respectively. Tyrosinase is a major rate-limiting enzyme that regulates melanin synthesis. Flavonoids have been shown to have inhibitory effects on melanin synthesis (Liu-Smith and Meyskens 2016). Arung et al. [23] reported that a prenylated flavonoid from the wood of *Artocarpus heterophyllus* had good activity against melanin synthesis through a reduction in tyrosinase activity. Worrawat et al. [24] isolated flavonoids from *Dalbergia parviflora* and showed that the flavonoids had inhibitory activity against murine tyrosinases and effectively inhibited melanin formation in B16 melanoma cells without being significantly toxic to the cells. These reports collectively suggest that SIFs extract could have good inhibitory activity against tyrosinase.

### 2.7. The effect of SIFs Extract on Antioxidant Enzymes Activities in A375 Cells

**Figure 5:**
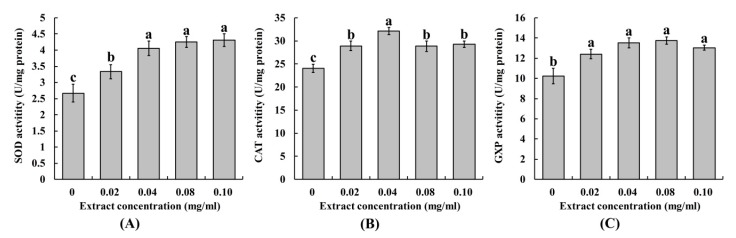
The effect of SIFs extract on the activities of antioxidant enzymes SOD (**A**), CAT (**B**), and GPX (**C**) in melanoma cells A375. Different small letters mean significant differences at *p* < 0.05. SOD: superoxide dismutase; GPX: glutathione peroxidase; CAT: catalase.

The antioxidant enzymes, such as catalase (CAT), glutathione peroxidase (GPX), and superoxide dismutase (SOD), are important for cells to maintain redox homeostasis. Cells can resist peroxidation damage by increasing the activity of these antioxidant enzymes. In melanocytes, α-MSH increase peroxide to promote melanin synthesis [25]. Antioxidants, like flavonoids, can regulate melanogenesis in melanocytes by regulating the antioxidant enzymes activities [26]. In the study, the activities of antioxidant enzymes (CAT, GPX, and SOD) in A375 cells were analyzed. The results showed that the activities of SOD, CAT, and GPX were increased in A375 cells when different SIFs extract concentrations were added (Figure 5). Therefore, it was hypothesized that SIFs could increase the activities of antioxidant enzymes (SOD, GPX and CAT) and thus inhibited the oxidative stress of A375 cells.

### 2.8. The Effect of SIFs Extract on Genes Related to the Melanin Synthesis Signaling Pathway

**Figure 6:**
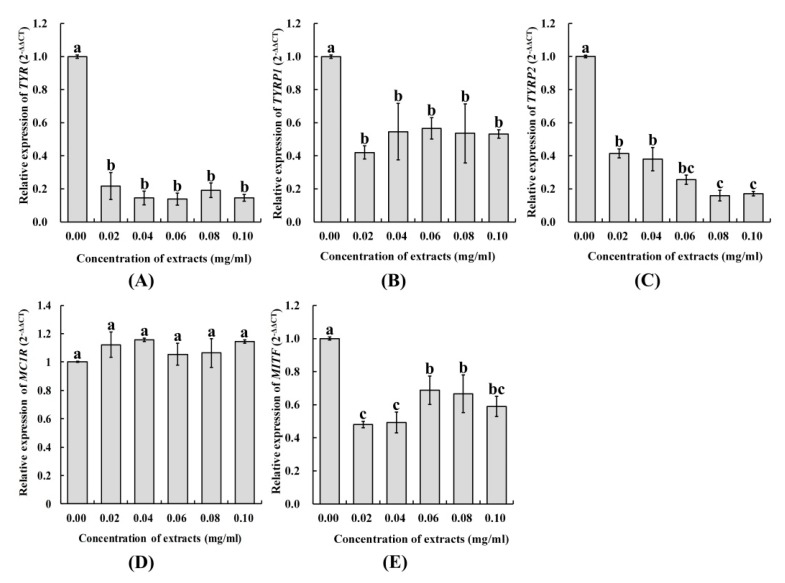
The effect of SIFs extract on relative expression of *TYR* (**A**), *TYRP1* (**B**), *TYRP2* (**C**), *MCIR* (**D**), and *MITF* (**E**) in melanoma cells A375.

α-MSH regulates melanin synthesis via a cAMP-dependent signaling pathway. When α-MSH binds to the melanocortin 1 receptor (MC1R), adenylate cyclase (AC) is activated by a G-protein-coupled receptor producing the intracellular second messenger, cyclic adenosine monophosphate (cAMP) [27]. The cAMP activates protein kinase (PKA), which phosphorylates the cAMP-response element binding protein (CREB) and activates gene expression of the microphthalmia-associated transcription factor (MITF) [28], further regulating genes related to melanin synthesis, such as tyrosinase (TYR), tyrosinase-related protein 1 (TYRP1), and tyrosinase-related protein 2 (TYRP2). Eventually, these enzymes promote the synthesis of intracellular melanin [29]. To study the effects of extract on melanin synthesis in A375 cells, we used qRT-PCR to detect genes related to melanin synthesis. Results showed that *TYP*, *TYPR1*, *TYPR2*, and MITF expression were downregulated, but that *MC1R* expression remained unchanged (Figure 6). Previous research reported that decreased MITF expression could be responsible for melanogenesis downregulation, which included decreases in TYP, TYPR1, and TYPR2 expression [30]. It was shown that SIFs extract could decrease melanin synthesis by inhibiting MITF expression and its associated regulating genes (TYR, TYPR1, and TYPR2) in A375 cells.

## 3. Materials and Methods 

### 3.1. Material

*S. involucrate* was bought from Wholesale market of medicinal materials, Xining, Qinghai Province. *S. involucrate* herbs were grounded into powder and used for ultrasound extraction experiment after overnight soaking.

### 3.2. Experimental Design

Single-factor experiments were used to preliminary study the different extraction process parameters, which including factors such as solvent ratio, ethanol content, and extraction time. The one factor was changed with the other factors remaining constant in each single factor experiment. The best processing of ultrasonic extraction of SIFs was determined by Box-Benhnken analysis method. For statistical calculations, the experimental variable *x_i_* was coded as *X_i_*, the equation was used as follows:

(2) $$X_{i}=\frac{x_{i}-x_{0}}{\Delta x}\left(i=1,\;2,\;3\right)$$

where *X_i_*, *x*_0_ and *Δx* are the dimensionless coded value of the variable, the value of *x_i_* at the center point, and the step change, respectively.

The experimental data were analyzed and processed for Equation (3) using Design Expert software including analysis of variance (ANOVA), which is suitable for experimental design.

(3) $$Y=\beta_{0}+\overset{3}{\underset{i=1}{\sum }}\beta_{i}X_{i}+\overset{3}{\underset{i=1}{\sum }}\beta_{ii}X_{i}^{2}+\overset{2}{\underset{i=1}{\sum }}\overset{3}{\underset{j=i+1}{\sum }}\beta_{ij}X_{i}X_{j}$$

where *Y* is the measured response value which associated with each factor level combination. *β_0_* is an intercept and *β_i_* is the regression coefficients computed from the observed experimental values of *Y,* and *X_i_* is the coded level of independent factor [31]. The *X_i_*, *X_j_* and $X_{i}^{2}$ are the interaction and quadratic terms, respectively.

The coefficient of determination R^2^, adjusted R^2^ and adequate precision were employed to evaluate the fit quality of the polynomial model equation. Model terms are chosen or rejected according to the probability of error (*p*) value with 95% confidence level. The polynomial equation fitted was expressed as 3D surface plots to visualize the relationship between the responses and the experimental level of each factor utilized in the design. The optimum region is also identified based on the main parameters in the overlay plot. The analysis of the data was accomplished by using the general factorial design of response surface methodology (RSM). The experimental conditions and results are displayed in Table 1 and Table 2.

### 3.3. Determination of Total Flavonoid Content

The determination method of total flavonoid content was referred to the method proposed of Jia et al. [32] with modifications. After added 0.3 mL of NaNO_2_ (5%, *w*/*v*) into 2 mL of samples, the mixture was allowed to stand at room temperature for 6 min. Then, 0.3 mL of Al(NO_3_)_3_ (10%, *w*/*v*) was added into this mixture and incubated for 10 min at room temperature. Finally, after 2 mL of NaOH (4%, *w*/*v*) added into this mixture, its absorbance was determined at 510 nm. The standard curve was prepared using various rutin concentrations as the standard. The SIFs content was calculated with the following equation:

(4) $$\mathrm{Total}\;\mathrm{flavonoids}\;\mathrm{content}\;\left(\%\right)=\frac{m\times V}{M}\times 100\%$$

where the *m* was total flavonoids in the 1 mL measured extracts, *V* was the total volume of extraction solution and M was the quality of the *S. involucrate* extracts.

### 3.4. Determination of Antioxidant Activities In Vitro

#### 3.4.1. Determination of Reducing Power

Ferrous ion reducing power was measured by using the method proposed of Sun et al. [33]. The different concentrations of extract (2.5 mL) were added into 2.5 mL of PBS (0.2 mg/mL, pH 6.6) and potassium ferricyanide (2.5 mL, 1%, *w*/*v*), followed by incubation at 50 °C for 20 min and then put into 2.5 mL of trichloroacetic acid (10%, *w*/*v*) to end the reaction. Finally, 2.5 mL of the supernatant was added 2.5 mL of deionized water and 0.5 mL of FeCl_3_ (0.1%, *w*/*v*). Then, it was plunged into darkness at 25 °C for 10 min, and the absorbance was determined at 700 nm. The L-ascorbic acid was used to prepare a standard solution for evaluating the reducing power. Each experiment was replicated for 3 times in the study.

#### 3.4.2. Determination of DPPH Scavenging Activity

DPPH scavenging ability was measured by using the method proposed of Qu et al. [34] with modifications. Different content of extract (1 mL) was added into 1 mL of 0.002 mg/mL DPPH (1.0 × 10^−4^ M in 50% ethanol). This mixture was measured at 517 nm after it incubated at room temperature in the dark for 30 min. The ascorbic acid was used as a positive control. Each experiment was replicated for 3 times in the study. The percentage of DPPH scavenging was calculated as the following equation:

(5) $$\;\mathrm{Scavenging}\;\mathrm{activity}\;\left(\%\right)=\left(1-\frac{A_{n}}{A_{0}}\right)\times 100\%\;\;\;\;\;\;\;\;\;\;\;\;$$

where *A_n_* is the absorbance of sample, and *A*_0_ is the absorbance of blank control solution without sample.

#### 3.4.3. Determination of ABTS^+^ Scavenging Activity

ABTS^+^ scavenging activity of SIFs extract was measured by using the method of Wang et al. [35] with slight modification. The 0.007 mg/mL ABTS^+^ was dissolved into 0.00245 mg/mL potassium persulphate solution. Then this mixture was incubated at 25 °C for 12 h in the dark. After diluted it with 75% of ethanol to adjust its absorbance to 0.70 *±* 0.02 at 734 nm. The 0.1 mL of different content of extract was put into 3.9 mL of ABTS^+^ solution. After incubating at room temperature for 10 min, the absorbance of this solution was measured at 734 nm. Each experiment was replicated for 3 times in the study. The calculation equation is consistent with the equation of DPPH scavenging activity.

#### 3.4.4. Determination of Hydroxyl Radical Scavenging Activity

The radical scavenging capability was measured by using the method of Mao et al. [36] with slight modification. Before added 2 mL of 0.006 mg/mL H_2_O_2_ into the mixture to start the reaction, the 2 mL of 0.006 mg/mL FeSO_4_ was added to 2 mL different concentrations of extract, and incubated this mixture at 37 °C for 30 min. Then 2 mL of 0.006 mg/mL salicylic acid was added into it, mixed, and incubated at 37 °C for 10 min. The absorbance was measured at 510 nm. Ascorbic acid was used as the reference compound for measuring the hydroxyl radical scavenging activity. Each experiment was replicated for 3 times in the study. The percentage of hydroxyl radical scavenging was calculated by the following equation:

(6) $$\mathrm{Scavenging}\;\mathrm{ability}\;\left(\%\right)=\left(1-\frac{A_{1}-A_{2}}{A_{0}}\right)\times 100\%\;\;\;\;\;\;\;\;\;\;\;\;\;\;\;$$

where *A*_1_ and *A*_2_ are the absorbance of a sample (with and without hydrogen peroxide, respectively), and *A*_0_ was the absorbance of distilled water.

#### 3.4.5. Determination of Superoxide Anion Scavenging Activity

The superoxide anion radical scavenging activity was measured using phenazine methyl sulfate (PMS), reduced coenzyme I (NADH), and nitro-blue tetrazolium (NBT) [37,38]. The 20 μL of NADH (2 mM) was added into the 180 μL of mixture which included 10 μL different content of extract, 20 μL NBT (1 mM), 20 μL PMS (0.1 mM), 40 μL potassium phosphate buffer (250 mM, pH 7.4) and 90 μL water. After this mixture was incubated for 20 min at room temperature, the discoloration was measured at 570 nm. Ascorbic acid was used as the reference compound. Each experiment was replicated for 3 times in the study, and the calculation equation of superoxide anion scavenging activity is consistent with the equation of DPPH scavenging activity.

### 3.5. Cell Experiment

#### 3.5.1. Cell Culture

Human melanoma A375 cells were bought from Kunming Institute of Kunming Institute of Zoology, Chinese Academy of Sciences (Kunming, China). The cells were cultured in DMEM which included 10% fetal bovine serum, and 1% of penicillin and streptomycin at 37 °C in a humidified atmosphere with 5% CO_2_.

Cell viability was detected by the colorimetric MTT method [39]. Cells were plated in 6-well plates (10^5^/well). Twenty-four hours after plating, extract (0–0.08 mg/mL) were added and cultures were incubated for an additional 24 h, each well was treated with 0.5 mg/mL MTT solution for 3 h at 37 °C. The resulting violet formazan precipitates were dissolved in DMSO and the absorbance of each well was detected at 590 nm using a microplate reader with a 630 nm reference.

#### 3.5.2. α-MSH Treatment

Human melanoma A375 cells were cultured for 24 h at a density of 10^5^ cells/mL in 6-well plates which contained 10% fetal bovine serum and 1% penicillin/streptomycin at 37 °C in a humidified atmosphere with 5% CO_2_. Then the medium was replaced by fresh one which included 100 nM α-MSH. These α-MSH-stimulated cells were used to all experiments treatment in next sections.

#### 3.5.3. Intracellular Tyrosinase Activity

After the α-MSH-stimulated cells (6-well, 10^5^ cells/well) incubated with different content of extract for 24 h, each well was washed and lysed with PBS (50 mM, pH 6.8) which contained 0.1 mM phenylmethyl-sulfonyl fluoride and 1% Triton X-100. The cells were disrupted by freezing and thawing, and lysates were clarified by centrifugation at 12,000 rpm for 30 min at 4 °C, the protein content of supernatant was determined by the Bradford method using BSA as standard. The supernatant was incubated in 1.25 mM L-DOPA and 25 mM PBS (pH 6.8) for for 1 h at 37 °C. The absorbance was measured at 475 nm [40]. The inhibition rate of tyrosinase activity was calculated by the following equation:

(7) $$\;\mathrm{Inhibition}\;\mathrm{rate}\;=\left(1-\frac{A_{1}-A_{0}}{A_{2}-A_{0}}\right)\times 100\%\;\;\;\;\;\;\;\;\;\;\;\;$$

where *A*_0_ is the absorbance control (without both test sample and α-MSH (L-DOPA alone)), *A*_1_ is the absorbance of reaction mixture containing test sample and α-MSH; *A*_2_ is the absorbance in the presence of the negative control (with α-MSH and without test sample).

#### 3.5.4. Melanin Content

The α-MSH-stimulated cells (6-well, 10^5^ cells/well) were incubated with different content of extract for 24 h. After being washed with PBS, a small number of cells were used to determine the protein content, and the rest of the cells were lysed with 1 mol NaOH and treated at 100 °C for 1 h. The cell lysates were clarified by centrifugation for 10 min at 10,000× *g.* Melanin contents were determined by the absorbance measured at 405 nm [40]. The inhibition rate of melanin synthesis activity was estimated as a percentage of the control culture.

#### 3.5.5. Determination of Gene Expression by qRT-PCR

The α-MSH-stimulated cells (2.5 × 10^5^ cells/well) were incubated with different content of extract for 24 h. Total RNA of cells in each well was extracted by Trizol (RNAiso Plus, Takara Biomedical Technology, Beijing, China). For the reverse transcription, 2 μg of total RNA was mixed with 1 μL of Oligo (dT)18 primer (Thermo Fisher Scientific, Waltham, MA, USA), 200 U of Revert Aid M-MuL Virus RT (Thermo) included 20 U of RiboLock RNase inhibitor (Thermo). After reverse transcription, 20 ng of cDNA was used to qPCR to amplify all genes in triplicate in a total reaction volume of 20 μL using GOTaq^®^ qPCR Master Mix (Promega, Madison, WI, USA), and the required amount of forward and reverse primers. Reactions were conducted on an LightCycler^®^ 96 (Roche Life Science, Penzberg, Germany) using the following cycling conditions: pre-incubation at 95 °C for 10 min, 3-step amplification at 95 °C for 10 sec, 60 °C for 30 sec, and 72 °C for 1 min. Expression of *β-actin* was used as an internal control for target gene expression. The genes and their sequences in melanin synthesis signaling pathway were from KEGG PATHWAY (map04916) and NCBI GenBank. Primers were designed by primer 5.0 software (Table 6). All primers were synthesized by Beijing Genomics Institute. The gene expression was calculated based on the 2^−ΔΔCt^ method [41].

#### 3.5.6. Detection of Superoxide Dismutase (SOD), Catalase (CAT) and Glutathione Peroxidase (GXP) Activities

The α-MSH-stimulated A375 cells (2.5 × 10^5^ cells/well) were incubated with different concentrations of the SIFs extract for 24 h, after which time the total protein was quantified using the BCA method; all results were fixed by the protein concentration [42,43].

To detect SOD activity, 100 μL of a 5 mg/mL NBT solution was placed into each well and cultured for 3 h. Then, 200 μL of dimethyl sulfoxide (DMSO) and 200 µL of 2 M NaOH were added dissolved in formazan blue. Absorbance was measured at 550 nm using a microplate reader. One unit of SOD activity was defined as the amount of enzyme that inhibited the formazan blue production rate per one hour [44].

For CAT activity, the cells were lysed into 200 μL of 1% Triton-X at 4 °C for 10 min. After centrifugation at 10,000 rpm for 10 min at 4 °C, the supernatant was collected in new tubes and mixed with 40 μL of 1 μM of H_2_O_2_. The absorbance was measured at 240 nm using a microplate reader. One unit of CAT activity was defined as the amount of enzyme that decomposed in 1 μM H_2_O_2_ per minute [45].

GPX activity was detected using the Sigma-Aldrich GPX Cellular Activity Assay Kit (Sigma-Aldrich, St. Louis, MO, USA) following the manufacturer’s instructions. One unit of GPX activity was defined as the amount of enzyme that oxidized 1 nM NADPH per minute, as measured by absorbance at 340 nm [46].

## 4. Conclusions

The optimum conditions for the ultrasonic extraction of SIFs were: extraction time of 64 min, an ethanol content of 54%, and a solvent ratio (*v*/*w*) of 54:1. The experimental value (4.89 ± 0.54%) was in agreement with the predicted value (4.80%). The extract of SIFs inhibited the oxidative stress and lipid peroxidation of A375 melanoma cells by increasing CAT, SOD, and GPX activities. In addition, SIFs extract could anti-deposition of melanin in human melanocytes by down-regulating genes (*TYP, TYPR1*, and *TYPR2*).

## Figures and Tables

**Table 1:** Level and code of independent variable used for response surface analysis.

Level	A: Extraction Time (min)	B: Ethanol Content (%)	C: Ratio of Liquor to Material (*v*/*w*)
−1	45	50	40
0	60	60	50
1	75	70	60

**Table 2:** Experimental design and results for response surface analysis. A, B, and C are extraction time, alcohol content, and ratio of liquor to material (*v*/*w*), respectively.

Run Order	Variable			Total Flavonoids Y (%)	
	A	B	C	Observed	Predicted
1	0	−1	1	4.59	4.42
2	−1	0	1	3.42	3.1325
3	0	−1	−1	3.81	3.42
4	0	0	0	4.38	4.23
5	1	−1	0	4.38	3.9425
6	0	0	0	4.68	4.23
7	1	1	0	3.24	2.7125
8	0	0	0	4.47	4.23
9	0	0	0	4.71	4.23
10	1	0	1	4.47	4.0325
11	−1	0	−1	3.78	3.4825
12	1	0	−1	3.33	2.8825
13	−1	−1	0	3.39	3.1625
14	−1	1	0	3.48	3.1925
15	0	0	0	4.77	4.23
16	0	1	−1	3.99	3.42
17	0	1	1	3.57	3.22

**Table 3:** The results of analysis on variance (ANOVA) for the effects of variables. A, B, and C are extraction time, alcohol content, and ratio of liquor to material (*v*/*w*), respectively.

Source	Sum of Squares	DF	Mean Square	F Value	*p*-Value Prob > F
Model	4.7575	9	0.5286	25.8661	0.0001
A	0.2278	1	0.2278	11.1474	0.0124
B	0.4465	1	0.4465	21.8489	0.0023
C	0.1624	1	0.1625	7.9490	0.0258
AB	0.3782	1	0.3782	18.5074	0.0036
AC	0.5625	1	0.5625	27.5244	0.0012
BC	0.3600	1	0.3600	17.6156	0.0041
A^2^	1.5654	1	1.5655	76.6011	<0.0001
B^2^	0.5756	1	0.5756	28.1675	0.0011
C^2^	0.2471	1	0.2471	12.0909	0.0103
Residual	0.1430	7	0.0204		
Lack of Fit	0.0303	3	0.0101	0.3594	0.7866
Pure Error	0.1127	4	0.0282		
Cor Total	4.9006	16			
DF = Degree of freedom					
Cor Total = Correlation Total					

**Table 4:** Regression coefficients estimate of the predicted quadratic polynomial model.

Factor	Coefficient Estimate	Standard Error
Constant	4.60	0.064
A	0.17	0.051
B	−0.24	0.051
C	0.14	0.051
AB	−0.31	0.071
AC	0.38	0.071
BC	−0.30	0.071
A^2^	−0.61	0.070
B^2^	−0.37	0.070
C^2^	−0.24	0.070

**Table 5:** EC_50_ and correlation analysis between antioxidant activities and content of extract and ascorbic acid.

	Extract			Ascorbic Acid		
	EC_50_ (mg/mL)	*p* Value	Multiple R	EC_50_ (mg/mL)	*p* Value	Multiple R
Ferrous Ion Reducing Power (A700)	0.69 ± 0.027	0.0609	0.976	0.032 ± 0.004	0.129	0.995
DPPH Scavenging Activity (%)	6.13 ± 0.97	0.000494	0.935	0.063 ± 0.007	0.0000215	0.990
ABTS Scavenging Activity (%)	0.88 ± 0.013	0.0220	0.945	0.065 ± 0.010	0.0000418	0.984
Hydrogen Peroxide Scavenging Activity (%)	1.80 ± 0.147	0.00161	0.995	0.11 ± 0.033	0.00955	0.996
Superoxide Anion Radical Scavenging Activity (%)	3.84 ± 0.368	0.00000486	0.989	0.27 ± 0.061	0.0343	0.997

**Table 6:** The primer sequences of genes in qRT-PCR.

Genes	RefSeq id	Sense Primer Sequence	Anti-Sense Primer Sequence
*TYR*	NM_000372.5	5′CACAGAGAGACGACTCTTGGTG3′	5′GCTGATGGTATGCTTTGCTAA3′
*TYRP1*	NM_000550.2	5′CCCCAGTCACCAACACAGAAA3′	5′CAGATAAGAAGCAGTCCCAAAA3′
*TYRP2*	NM_001129889.2	5′TATTAGGACCAGGACGCCC3′	5′CATCCAAGCTATCACAGACAGT3′
*MITF*	NM_000248.3	5′GAGAACAGCAACGCGCAAAAG3′	5′CAGTGACACCGACGGGAGAAA3′
*MC1R*	NM_002386.3	5′GTCAAAGAGGATGGACTAAATGATC3′	5′CAGGAGTGGGCGGAAAA3′
*β-actin*	NM_001101.5	5′TTGCGTTACACCCTTTCTTG3′	5′TCACCTTCACCGTTCCAGT3′

## Supplementary Materials

The following are available online at https://www.mdpi.com/1420-3049/25/2/313/s1.

## Abbreviations

ABTS—2,2-azino-bis-3-ethylbenzthiazoline-6-sulphonic acid; BBD—Box-Behnken design; DPPH—2, 2-diphenyl-1-picrylhydrazyl; RSM—Response surface methodology; SIFs—*Saussurea involucrate* flavonoids; CAT—Catalase; GPX—Glutathione peroxidase; SOD—Superoxide Dismutase
