# Supplementary material for: Optimization of Ultrasonic Flavonoid Extraction from Saussurea involucrate, and the Ability of Flavonoids to Block Melanin Deposition in Human Melanocytes
Source: Molecules. 2020 Jan 13;25(2):313. doi: 10.3390/molecules25020313 (PMC7024147; doi:10.3390/molecules25020313)
Supplement: Supplementary file 1 [file molecules-25-00313-s001.pdf]

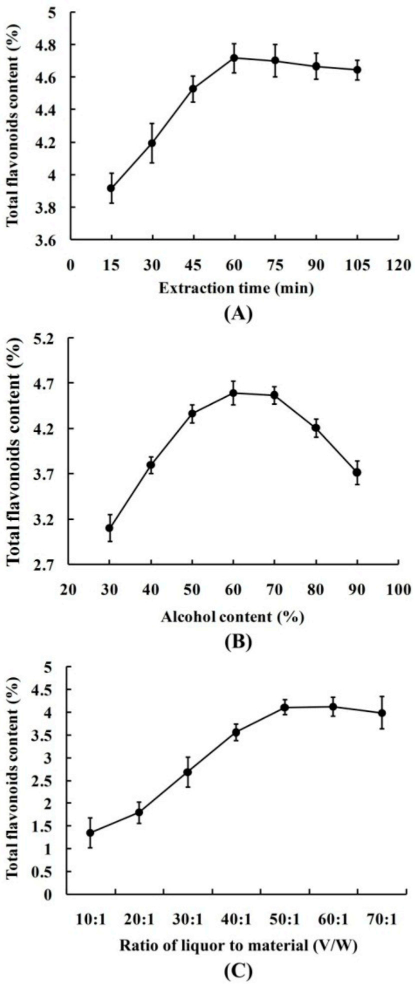

Supplementary figure 1. The single factor experiments. A: extraction time (min), B: ethanol content (%), and C: ratio of liquor to material (V/W)
